# Perinatal Characteristics and the Sensitization to Cow Milk, Egg Whites and Wheat in Children up to 3 Years of Age

**DOI:** 10.3390/children10050860

**Published:** 2023-05-11

**Authors:** Hsin-Yu Chang, Zon-Min Lee, Ling-Sai Chang, Wei-Ling Feng, Yao-Hsu Yang, Mei-Chen Ou-Yang

**Affiliations:** 1 Department of Pediatrics, Kaohsiung Chang Gung Memorial Hospital, Kaohsiung 833, Taiwan; 2 College of Medicine, Chang Gung University, Taoyuan 333, Taiwan; 3 Department of Pharmacy, Kaohsiung Chang Gung Memorial Hospital, Kaohsiung 833, Taiwan; 4 Department of Pharmacy, Tajen University, Pingtung 907, Taiwan; 5 The Biostatistics Center, Kaohsiung Chang Gung Memorial Hospital, Kaohsiung 833, Taiwan; 6 Health Information and Epidemiology Laboratory, Chiayi Chang Gung Memorial Hospital, Chiayi 613, Taiwan; 7 Department of Traditional Chinese Medicine, Chiayi Chang Gung Memorial Hospital, Chiayi 613, Taiwan; 8 School of Traditional Chinese Medicine, College of Medicine, Chang Gung University, Taoyuan 333, Taiwan

**Keywords:** Chang Gung Research Database, cow milk, egg whites, specific immunoglobulin E, wheat

## Abstract

Food sensitization in early life identifies children at risk of developing allergic diseases. We investigated the sensitization to cow milk (CM), egg whites, and wheat. Newborns and infants under 3 years of age with available specific immunoglobulin E (sIgE) data were identified. A retrospective survey was conducted using data from the Chang Gung Research Database. Perinatal characteristics, such as singleton or multiples in a single pregnancy, parity, meconium staining, maternal age, spontaneous delivery or cesarean section, meconium passage, weeks of gestation, birth length, body weight, head and chest circumferences, and season, were obtained. The data on sIgE were collected, and a logistic regression model was used to determine the odds of sensitization. Positive sIgE for CM and egg whites was more likely to occur in boys than in girls. Early-life egg white and wheat sensitization was associated with increased birth body length and weight. A multivariate analysis indicated an association between egg white sIgE positivity and logarithmic total IgE. Higher total IgE levels and younger age were associated with egg white sensitization, and elevated weight and length at birth were linked to food sensitization, particularly to egg whites and wheat.

## 1. Introduction

Early-life food sensitization has identified children who are at high risk for subsequent allergic diseases [1]. In particular, milk, eggs, and wheat were the most common food hypersensitivities encountered during the first 3 years of life [2]. A prospective birth cohort study addressed prenatal and perinatal issues and identified risk factors for food allergy in Taiwanese young children [3]. The survey demonstrated that eggs (26/95, 27.3%) and milk (22/95, 23.2%) were the most common food allergens.

Food allergies are complex diseases characterized by genetic and environmental factors [4,5]. Cow milk (CM) was often the first food introduced into a diet after breast milk. In a study on CM allergy (CMA), a European birth cohort of 12,049 children showed 0.54% challenge-proven CMA and 23.6% non-immunoglobulin (Ig) E-mediated CMA [6]. Children showed the maximum response of positive specific (s)IgE to CM (80%) [7]. One factor associated with the development of child-onset food allergies was a higher level of total IgE than that in adult-onset allergies [7]. Evidence from a randomized clinical trial of 330 enrolled neonates at risk of atopy suggested that sensitization to CM and food allergies, including CMA and anaphylaxis, risk of asthma, and recurrent wheezing in young children, especially among those with high total IgE levels, was primarily prevented by avoiding CM formula supplementation at least for the first 3 days of life [8,9]. However, the introduction of small amounts of CM between 1 and 2 months of life was associated with a lower likelihood of CMA [10]. Of all children with confirmed IgE-mediated CMA, fewer tolerated CM 1 year after diagnosis than infants with negative sIgE. Boer et al. identified cut-offs for milk sIgE in relation to baked milk tolerance that could support timely decisions regarding referrals to an oral food challenge [11]. Laboratory tests assisted in diagnosing IgE-mediated CMA. Early diagnosis of CMA is important because delayed diagnosis may lead to nutritional disorders and a subsequent increased risk of impaired growth [12]. Egg white allergies are another common food allergy that frequently occurs in the first year of life. These allergies could be immediate (IgE-mediated) or delayed (non-IgE-mediated) and could involve many organ systems [13]. A prospective database revealed that nearly one-third of the infants were allergic to multiple foods, including the three main allergens in Japan: egg, milk, and wheat [14,15]. However, some of the evidence about the perinatal factors for food sensitization was conflicting [16]. This study aimed to provide a better understanding of the perinatal characteristics associated with the odds of sensitization to CM, egg whites, and wheat.

## 2. Methods

**Figure 1:**
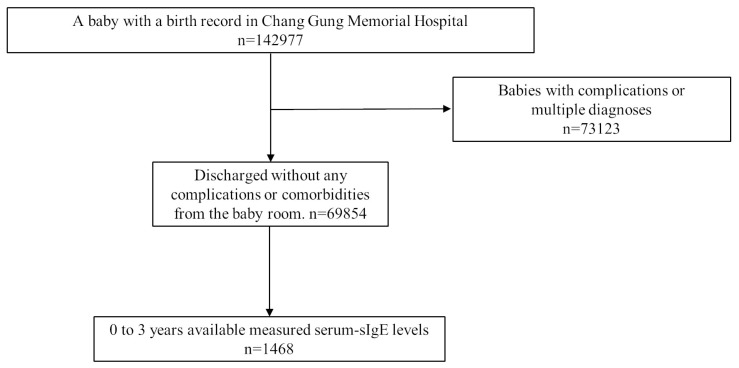
Schematic explanation of the enrolled cohort. sIgE, specific immunoglobulin E.

This retrospective study used data from the Chang Gung Research Database to investigate the association between perinatal characteristics and sIgE against CM, egg whites, and wheat from 2015 to 2019. The research protocol was approved by the Ethics Committee of the Chang Gung Memorial Hospital (202001016B0). As shown in Figure 1, we included children aged 0–3 years, selected from patients who had available measured serum-sIgE levels and who were healthy term neonates admitted to the baby room (n = 1468). Our inclusion criteria were babies with a single diagnosis of live-born infants (the International Classification of Disease, 9th Revision Clinical Modification, ICD-9-CM V30–37 or ICD-10-CM Z38) at the time of discharge from the baby room, normal spontaneous delivery, or cesarean section as per the payment category in the basic file of the hospitalization. They were considered discharged without any complications or comorbidities. We investigated CM (n = 1100), egg white (n = 1098), and wheat (n = 1089) sensitization. Children with allergic symptoms suspected of having food allergies were tested, and we identified the phenotypes of asthma/wheezing, dermatitis, and rhinitis using the codes provided by ICD-9-CM prior to 2016 and ICD-10 afterward. Data were extracted from the database of patients tested using the ImmunoCAP (Thermo Fisher Scientific Inc., Phadia AB, Uppsala, Sweden) or a multiple allergen simultaneous test system (MAST, OPTIGEN; Hitachi Chemical Diagnostics, Inc., Mountain View, CA, USA). Total IgE was detected using ImmunoCAP or nephelometry at the time of a specific IgE survey [17].

Specific IgE between 0 and 0.34 kUA/L using ImmunoCAP or 0 and 26 LUs using MAST was considered negative [18,19]. For ImmunoCAP, positive sensitization (class 1–6) referred to values > = 0.35 kUA/L, and for MAST it referred to values > = 27 LUs [20,21]. Associated factors included singleton or multiple pregnancies, parity, meconium staining, maternal age, normal spontaneous delivery or cesarean section, birth length, body weight, head and chest circumferences, meconium passage, birth season, and weeks of gestation. Mothers who underwent emergency cesarean section for premature birth with tocolytic failure, babies with fetal distress, or severe maternal diseases such that newborns could not be admitted to the baby room were excluded from this study [22,23]. We performed a sensitivity analysis due to the increasing numbers of food allergy diagnoses after Learning Early About Peanut Allergy (LEAP) trial on evaluated allergens [24].

Categorical results were presented as numbers and continuous results as means ± standard deviation (SD) or median (range). We used chi-square or the Fisher’s exact test to detect significant differences between sensitization to CM, egg whites, or wheat, and characteristics, such as sex, mode of delivery, parity, birth season, maternal age, and other perinatal factors. Student *t*-test was applied to explore the effect of gestational age, birth weight/length, and maternal ages divided into two groups (< 40 years and ≧ 40 years). One-way analysis of variance was used to compare the three groups of maternal age (≦ 25 years, 25–40 years, and ≧ 40 years). Odds ratios (ORs) were adjusted for confounding variables and calculated using negative sensitization as the reference category. Adjustments with multivariable logistic regression were allowed for possible relevant confounders and factors showing a potential influence in univariate analysis, such as age, sex, logarithmic (log) total IgE, and birth body length, to identify the independent factors for allergen sensitization in infants. Analyses were performed using the Statistical Analysis System Package (SAS statistical software version 9.4; SAS Institute, Cary, NC, USA).

## 3. Results

### 3.1. Is There a Role of Perinatal Characteristics for sIgE of CM?

**Figure 2:**
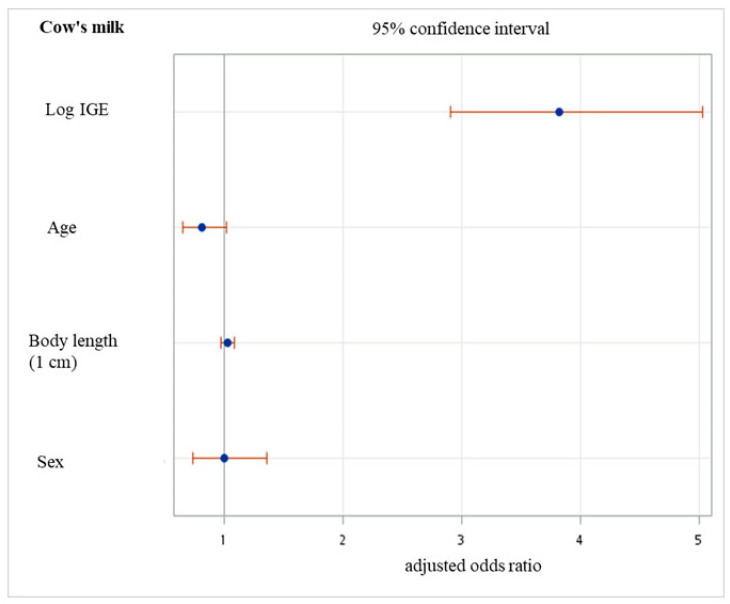

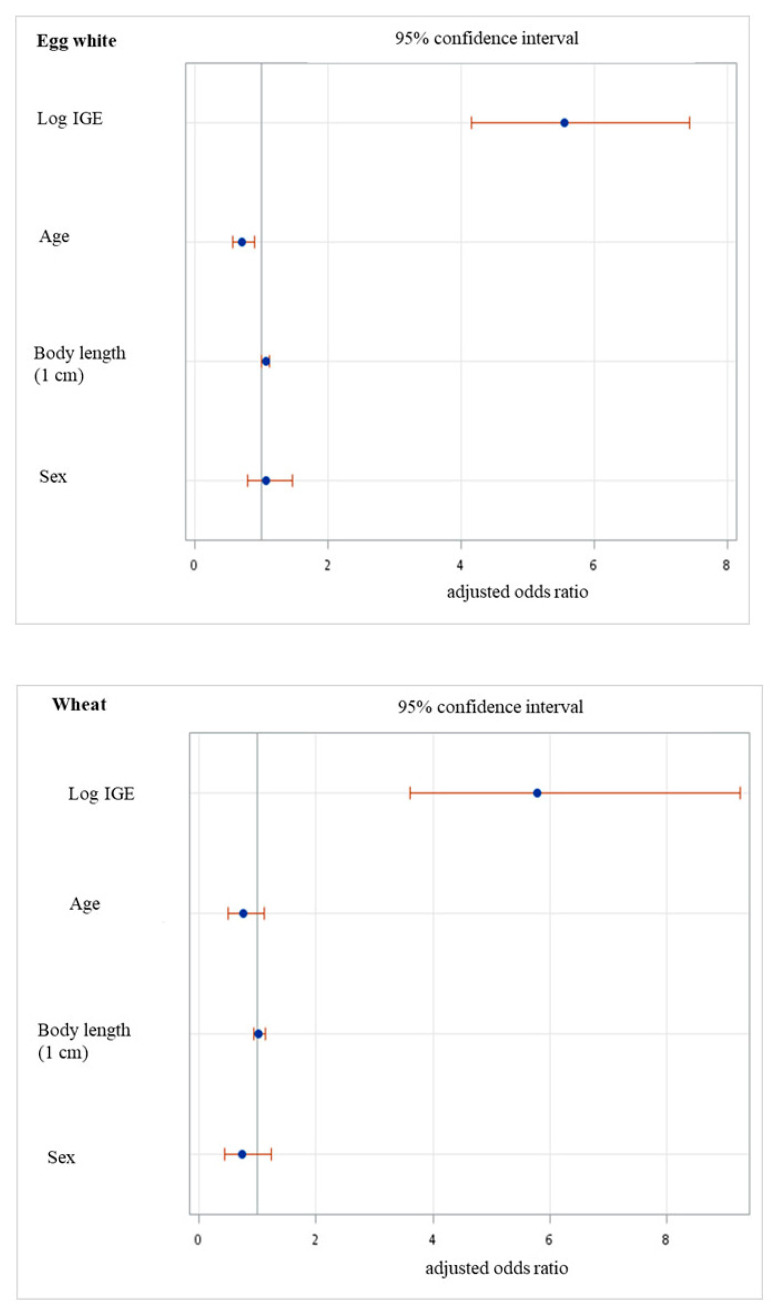
Multiple logistic regression model for sensitization. IgE, immunoglobulin E; Log, logarithmic.

Among the 1,100 children with available CM-sIgE, 307 (27.9%) tested positive for the sIgEs against CM. In total, 32.1% and 8.2% of the children tested positive for egg whites- and wheat-sIgE, respectively (Table 1). A total of 27 children were sensitized to eggs and wheat, 147 to CM and eggs, 2 to CM and wheat, and 54 to all three foods. The mean age at diagnosis of positive sIgE to CM was 2.0 years (SD, 0.6 years). Positive CM-sIgE levels were more likely to occur in boys (*p* = 0.012; Table 1). No significant difference in age was observed in the negative and positive groups (2.0 ± 0.7 and 2.0 ± 0.6 years, *p* = 0.286). The children with positive sIgE to CM had higher total serum IgE levels than the children with negative sIgE (median 178 U/mL (range 11.9–3749.0) in the group with positive sIgE; 59.2 U/mL (range 2.1–4349.0) in the group with negative sIgE; *p* < 0.001 of both log and arithmetic calculations). We found significantly higher log total IgE levels in boys when we divided our data into groups with negative and positive CM-sIgE (log total IgE 1.9 ± 0.6 in the boys’ negative group; 1.7 ± 0.6 in the girls’ negative group, *p* < 0.001; 2.3 ± 0.5 in the boys’ positive group, 2.2 ± 0.5 in the girls’ positive group, *p* = 0.046) [25]. The mean gestational age of the patients in the negative sensitization group was 38.5 (SD ± 1.6) weeks and that in the positive group was 38.5 (SD ± 1.3) weeks (*p* = 0.613). We hypothesized that there was an association between perinatal characteristics and the odds of sensitization to CM and found that CM-positive sIgE was not associated with cesarean delivery, number of fetuses, parity, meconium staining, maternal age, birth length and weight, head and chest circumferences, meconium passage, or birth season (*p* > 0.05). Similar results were observed when the patients were grouped according to sex. Independent factors for sIgE positive to CM were elevated total IgE or log total IgE values (adjusted odds ratio (aOR) 1.001, *p* < 0.001; aOR 3.820, *p* < 0.001) and not age, birth body length, or sex (Figure 2).

### 3.2. Is There an Effect of Perinatal Characteristics on sIgE of Egg White and Wheat?

Our findings indicate that a greater proportion of boys (36.3%) than girls (26.4%) aged 0–3 years had egg white sensitization (*p* = 0.001, Table 1). Age and gestational age were similar in the two groups with negative and positive egg white-sIgE. The mean age of allergic children with negative egg white-sIgE was 2.0 years (SD 0.7), and that of children with positive sIgE was 2.0 years (SD 0.7, *p* = 0.207). The mean levels (SD) of gestational age in children with negative and positive egg white-sIgE were 38.5 ± 1.6 weeks and 38.5 ± 1.3 weeks (*p* = 0.629), respectively. Two hundred and seventy-six (25.1%) children were born by a cesarean section (*p* = 0.796 between the groups with negative and positive egg white-sIgE). Furthermore, greater birth body lengths and weights were associated with significantly higher odds of egg white and wheat IgE positivity (*p* < 0.05, Table 2A, Table 2B and Table 2C). This association between birth body length and egg white-sIgE persisted even after controlling for age, sex, and log total IgE (adjusted OR (aOR), 1.067, 95% confidence interval (CI), 1.007–1.130, *p* = 0.029, Figure 2). The independent factors for positive sIgE to egg whites were younger age and higher log total IgE (aOR 0.713, 95% CI, 0.568–0.896, *p* = 0.004; aOR 5.556, 95% CI, 4.151–7.437, *p* < 0.001) but not sex (aOR 1.076, 95% CI, 0.791–1.465, *p* = 0.641). 

### 3.3. Perinatal Characteristics and the Odds of Sensitization

Singleton or multiple pregnancies, parity, meconium staining, maternal age, normal spontaneous delivery or cesarean section, birth head and chest circumferences, meconium passage, birth season, and weeks of gestation were not associated with egg white sensitization. A univariate analysis showed that the developments of sIgE to CM, egg whites, or wheat were not related to birth season (*p* > 0.05). Maternal age was similar in the negative and positive groups whether we divided it into two groups (<40 years and ≧40 years, Table 2A, Table 2B and Table 2C) or three groups (≦25 years, 25–40 years, and ≧40 years; *p* > 0.05). 

### 3.4. Association between Allergic Diseases and Sensitization to Allergens

In this association analysis, 53.4% of the patients with positive CM-sIgE had significantly higher rates of asthma or wheezing than patients with negative CM-sIgE (38.0%, *p* = 0.006 after the Bonferroni correction for multiple comparisons) but not wheat (*p* = 1.000; Table 3). Children with positive CM sensitization had a significantly increased coexistence of asthma and rhinitis (*p* = 0.014). Children with wheat sensitization had similar odds of developing the above-mentioned allergic diseases compared to those without wheat sensitization (*p* > 0.05) (Table 3).

### 3.5. Effect of LEAP Trial on Evaluated Allergens

Stratification analysis of the multiple logistic regression model showed consistent results in the group with CMA before 2015 and after 2016 (aOR of log total IgE 1.003, 95% CI 1.001–1.004, *p* = 0.003; aOR 1.001, 95% CI 1.000–1.001, *p* < 0.0001; Table 4A). In the group with egg white allergy, birth body length and log total IgE were significantly associated with the odds after 2016 (aOR of birth body length, 1.083, 95% CI 1.018–1.152, *p* = 0.012; aOR of log total IgE, 1.002; 95% CI 1.002–1.003, *p* < 0.0001; Table 4B). Analysis of patients enrolled for wheat tests after 2016 showed that log total IgE had significantly associated odds (aOR, 1.001; 95% CI 1.001–1.001, *p* < 0.0001); however, this was not significant before 2015 (aOR, 1.001; 95% CI 0.999–1.002, *p* = 0.306; Table 4C).

## 4. Discussion

This study investigated the association between perinatal factors and IgE sensitization to common food allergens. This retrospective survey, which involved a large database, enhanced the findings of previous studies. The results showed a relationship between sIgE against egg whites and log total IgE, younger age, and birth body length. These findings agreed with the results of previous studies that tested egg white tolerance and indicated that most egg allergies developed in the first year of life and that the overall prognosis for tolerance was good [14,26,27]. Healthy newborns with larger body sizes tended to develop food sensitization, especially for egg whites and wheat. In accordance with the findings of a previous longitudinal study, which found that a decreasing trend in infantile body mass index Z-scores was significantly associated with a higher prevalence of IgE sensitization in 1-year-olds, a previous cross-sectional study revealed that body height was negatively associated with egg white sensitization and positively associated with mite sensitization in a preschool subgroup of children with allergies [28,29]. An increased surface area of contact with the allergens was suspected. The study of atopic phenotypes and skin prick tests of inhaled allergens identified no association between birth body length and atopy. However, our study focused on food and not inhalant allergens [30]. Future research should investigate the longitudinal changes in body sizes, including body height and weight.

The landmark LEAP trial suggested that peanut allergies could be prevented through early peanut introduction after screening high-risk infants. These guidelines have been rewritten to provide strong evidence that early peanut crop introduction has a potentially dramatic benefit. Multiallergen testing in infants became more popular after the LEAP study [24,31,32,33]. The findings agreed well with the subgroup results of our study (Table 4A, Table 4B and Table 4C).

The relevant literature on male predisposition to allergic diseases or allergens appears to be compatible with the specific results obtained in the present study [34,35]. Numerous studies in the literature have dealt with the role of sex in total IgE concentration and suggested significantly higher total IgE levels in male patients, for both children and adults [36]. One explanation for this finding was sex differences in the stress response systems to early-life stress and the association between increased prenatal maternal stress and early asthma phenotypes [37]. In contrast, certain food allergies, such as shrimp, demonstrated no sex-related differences [38]. In line with our results, these findings suggest that the effects of cesarean section, gestational age, multiparity, birth season, and maternal age were not pronounced among children with food allergies [39,40,41,42]. Regardless of food types, studies have indicated that children born in autumn and winter were more prone to food allergies, but Taiwan has plenty of sunshine throughout the year and thus, has little effect on the fluctuation of vitamin D [43,44].

A nationwide Swedish cohort study found that food allergies were more likely to occur in girls and were associated with cesarean delivery during a 13-year follow-up period. However, the above-mentioned study did not exclude newborns with complications, such as premature birth, nor did it analyze specific food allergies, and it was difficult to believe that perinatal factors still had an impact on food allergies 13 years later [39]. In a birth cohort study, cesarean sections were reported to cause a predisposition to food allergies [40]. 

Patients included in this analysis were highly atopic. These findings are in accordance with previous results that tested highly atopic infants with greater sensitization to CM, egg whites, and wheat, even though these studies used different measures of sIgE [45]. Although CM sensitization was frequently observed in admitted wheezing infants, positive CM-sIgE was not predictive of later childhood asthma induced by wheat, egg white, or inhalation allergens [46,47,48]. While fetal measurements are a biologically plausible index of respiratory function, whether positivity for egg white is significantly correlated with birth body length remains unclear [49].

The strengths of this study included the large database design and detailed perinatal stress factor analysis (i.e., previously identified vulnerable developmental windows for atopy risk and stress programming). Despite the large number of participants in the database study, the present study had some limitations. The first limitation was rooted in false negatives, which were non-IgE-mediated food allergies [39,50]. The second limitation was that children with allergy symptoms were more likely to be tested, and our patients were those who tended to have high total IgE, resulting in selection bias and a high positive rate. Although the current study used a large database design with detailed perinatal factors, some information influencing food sensitization could not be acquired from the database, including breastfeeding duration, maternal smoking, pet exposure, maternal supplementation of vitamin D or folate, timing of solid food introduction, day care attendance, and family history of allergy [51]. After validation, the Chang Gung Research Database included a population with high disease severity [52]. The adults did not visit medical centers for mild rhinitis or dermatitis [53]. We also found that the positive rate of food allergens in adults was much lower than that in children [54]. Therefore, maternal sIgE levels and reported maternal allergic diseases were not included in the analysis of this database.

However, the influence of birth factors on food sIgE levels remains controversial. In summary, higher total IgE levels and a younger age were associated with egg white sensitization. We found that increased birth weight and length were associated with food sensitization, particularly to egg whites and wheat. 

## Figures and Tables

**Table 1:** Participant characteristics.

M ± SD or n (%)	Negative sIgE to Milk	Positive sIgE to Milk	*p*
All (n)	793 (72.1%)	307 (27.9%)	
Age at sIgE tests	2.0 ± 0.7	2.0 ± 0.6	0.286
Sex of child			0.012 *
Male	443	197	
Female	350	110	
M ± SD or n (%)	Negative sIgE to egg	Positive sIgE to egg	*p*
All (n)	745 (67.9%)	353 (32.1%)	
Age at sIgE tests	2.0 ± 0.7	2.0 ± 0.7	0.207
Sex of child			0.001 *
Male	407	232	
Female	338	121	
M ± SD or n (%)	Negative sIgE to wheat	Positive sIgE to wheat	*p*
All (n)	1000 (91.8%)	89 (8.2%)	
Age at sIgE tests	2.0 ± 0.7	2.1 ± 0.7	0.241
Sex of child			0.442
Male	576	55	
Female	424	34	

* Significant, *p* < 0.05; M ± SD, mean ± standard deviation; sIgE, specific immunoglobulin E.

**Table 2A:** Perinatal characteristics and the sensitization to cow milk.

M ± SD or n (%)	Negative sIgE to Milk	Positive sIgE to Milk	*p*
Gestational age (weeks)	38.5 ± 1.6	38.5 ± 1.3	0.613
Birth weight (g)	3013.9 ± 547.2	3076.3 ± 514.9	0.085
Birth length (cm)	48.9 ± 3.0	49.3 ± 2.8	0.063
Birth head circumference (cm)	33.4 ± 1.8	33.6 ± 1.7	0.139
Birth chest circumference (cm)	32.0 ± 2.3	32.2 ± 2.1	0.061
Maternal age at birth (years)			0.748
<40	623	231	
≧40	70	28	
Number of fetuses:			0.764
Single	765	295	
Multiple	28	12	
Parity			0.443
First	777	300	
Second or third	16	7	
Meconium stain			0.687
Negative	717	280	
Positive	76	27	
Meconium passage			0.690
Negative	493	189	
Positive	299	117	
Season of birth			0.211
Spring	162	79	
Summer	205	79	
Fall	221	72	
Winter	205	77	
Serum log total IgE (U/mL)	1.8 ± 0.6	2.3 ± 0.5	<0.001 *
Delivery method			0.217
Vaginal delivery	602	222	
Cesarean delivery	191	85	

^*^ Significant *p* value < 0.05; cm, centimeter; M ± SD, mean ± standard deviation; log, logarithmic; sIgE, specific immunoglobulin E; spring, March, April, and May; summer, June, July, and August, autumn, September, October, and November; and winter, December, January, and February.

**Table 2B:** Perinatal characteristics and the sensitization to egg whites.

M ± SD or n (%)	Negative sIgE to Egg	Positive sIgE to Egg	*p*
Gestational age (weeks)	38.5 ± 1.6	38.5 ± 1.3	0.629
Birth weight (g)	2998.2 ± 562.0	3100.9 ± 480.8	0.002 *
Birth length (cm)	48.8 ± 3.1	49.5 ± 2.6	<0.001 *
Birth head circumference (cm)	33.4 ± 1.9	33.6 ± 1.6	0.066
Birth chest circumference (cm)	32.0 ± 2.4	32.2 ± 2.0	0.096
Maternal age at birth (years)			0.477
<40	578	274	
≧40	63	35	
Number of fetuses:			0.324
Single	715	343	
Multiple	30	10	
Parity			0.129
First	727	348	
Second or third	18	5	
Meconium stain			0.788
Negative	677	319	
Positive	68	34	
Meconium passage			0.689
Negative	468	214	
Positive	276	138	
Season of birth			0.670
Spring	155	85	
Summer	194	90	
Fall	202	90	
Winter	194	88	
Serum log total IgE (U/mL)	1.7 ± 0.6	2.3 ± 0.5	<0.001 *
Delivery method:			0.796
Vaginal delivery	556	266	
Cesarean delivery	189	87	

* Significant *p* value < 0.05; cm, centimeter; M ± SD, mean ± standard deviation; log, logarithmic; sIgE, specific immunoglobulin E; spring, March, April, and May; summer, June, July, and August, autumn, September, October, and November; and winter, December, January, and February.

**Table 2C:** Perinatal characteristics and the sensitization to wheat.

M ± SD or n (%)	Negative sIgE to Wheat	Positive sIgE to Wheat	*p*
Gestational age (weeks)	38.5 ± 1.5	38.7 ± 1.1	0.197
Birth weight (g)	3016.9 ± 548.3	3154.1 ± 413.9	0.004 *
Birth length (cm)	48.9 ± 3.0	49.5 ± 2.3	0.025 *
Birth head circumference (cm)	33.4 ± 1.8	33.7 ± 1.4	0.071
Birth chest circumference (cm)	32.0 ± 2.3	32.5 ± 1.8	0.015 *
Maternal age at birth (years)			0.523
<40	775	71	
≧40	87	10	
Number of fetuses			0.182
Single	961	88	
Multiple	39	1	
Parity			0.352
First	977	89	
Second or third	23	0	
Meconium stain			0.632
Negative	906	82	
Positive	94	7	
Meconium passage			0.909
Negative	622	56	
Positive	376	33	
Season of birth			0.516
Spring	226	14	
Summer	256	24	
Fall	265	26	
Winter	253	25	
Serum log total IgE (U/mL)	1.9 ± 0.6	2.5 ± 0.5	<0.001 *
Delivery method:			0.920
Vaginal delivery	748	67	
Cesarean delivery	252	22	

* Significant *p* value < 0.05; cm, centimeter; M ± SD, mean ± standard deviation; log, logarithmic; sIgE, specific immunoglobulin E; spring, March, April, and May; summer, June, July, and August, autumn, September, October, and November; and winter, December, January, and February.

**Table 3:** Association between allergic diseases and sensitization to allergens.

n (%)	Negative sIgE to Milk	Positive sIgE to Milk	*p* ^a^	Negative sIgE to Egg	Positive sIgE to Egg	*p* ^a^	Negative sIgE to Wheat	Positive sIgE to Wheat	*p* ^a^
All (n)	793 (72.1%)	307 (27.9%)		745 (67.9%)	353 (32.1%)		1000 (91.8%)	89 (8.2%)	
Asthma			0.006 *			0.154			1.000
Negative	492	143		447	186		579	47	
Positive	301	164		298	167		421	42	
Dermatitis			0.014 *			1.000			1.000
Negative	493	222		490	224		647	55	
Positive	300	85		255	129		353	34	
Rhinitis			1.000			0.287			1.000
Negative	365	136		355	145		462	38	
Positive	428	171		390	208		538	51	
Asthma and dermatitis			1.000			0.212			1.000
Negative	730	275		690	313		915	78	
Positive	63	32		55	40		85	11	
Asthma and rhinitis			0.014 *			0.273			1.000
Negative	594	202		553	241		725	62	
Positive	199	105		192	112		275	27	
Dermatitis and rhinitis			1.000			0.126			1.000
Negative	660	263		639	283		840	72	
Positive	133	44		106	70		160	17	
Asthma, dermatitis, and rhinitis			1.000			0.210			1.000
Negative	757	288		715	328		951	83	
Positive	36	19		30	25		49	6	

N, number. ^a^ After the Bonferroni correction for multiple comparisons * Significant *p*-value < 0.05; sIgE, specific immunoglobulin E.

**Table 4A:** Subgroup analyses to evaluate effects of Learning Early About Peanut Allergy trial: milk sensitization.

2015 and before 2015 (n = 171)	Adjusted Odds Ratio	95% Confidence Interval	*p*
Log total IgE	1.003	1.001–1.004	0.003 *
Age	1.165	0.659–2.06	0.598
Birth body length	1.08	0.955–1.222	0.222
Sex	1.415	0.665–3.012	0.368
**2016 and after 2016 (n = 816)**			
Log total IgE	1.001	1.000–1.001	<0.0001 *
Age	0.971	0.775–1.216	0.797
Birth body length	1.026	0.968–1.087	0.385
Sex	1.163	0.844–1.601	0.357

* Significant *p*-value < 0.05; IgE, immunoglobulin E; log, logarithmic.

**Table 4B:** Subgroup analyses to evaluate effects of Learning Early About Peanut Allergy trial: egg whites sensitization.

2015 and before 2015 (n = 170)	Adjusted Odds Ratio	95% Confidence Interval	*p*
Log total IgE	1.001	1.000–1.002	0.039 *
Age	1.178	0.678–2.046	0.561
Birth body length	1.073	0.954–1.208	0.241
Sex	1.331	0.639–2.774	0.445
**2016 and after 2016 (n = 816)**			
Log total IgE	1.002	1.002–1.003	<0.0001 *
Age	0.851	0.681–1.063	0.156
Birth body length	1.083	1.018–1.152	0.012 *
Sex	1.23	0.893–1.693	0.205

* Significant *p*-value < 0.05; IgE, immunoglobulin E; log, logarithmic.

**Table 4C:** Subgroup analyses to evaluate effects of Learning Early About Peanut Allergy trial: wheat sensitization.

2015 and before 2015 (n = 167)	Adjusted Odds Ratio	95% Confidence Interval	*p*
Log total IgE	1.001	0.999–1.002	0.306
Age	0.852	0.336–2.157	0.735
Birth body length	1.135	0.904–1.425	0.274
Sex	0.963	0.282–3.286	0.952
**2016 and after 2016 (n = 809)**			
Log total IgE	1.001	1.001–1.001	<0.0001 *
Age	1.033	0.696–1.532	0.872
Birth body length	1.029	0.931–1.137	0.580
Sex	0.955	0.556–1.642	0.869

* Significant *p*-value < 0.05; IgE, immunoglobulin E; log, logarithmic.

## Data Availability

Data is unavailable because the owner of this database is Chang Gung Memorial Hospital.
